# Differential Plasma Expression of sTNF-R, TNF-α, PDGF-AA, IL-17A, and IL-1β Across the Colorectal Neoplasia Spectrum

**DOI:** 10.3390/biom16030426

**Published:** 2026-03-13

**Authors:** Vlad-Alexandru Ionescu, Gina Gheorghe, Claudiu Stefan Turculet, Teodor Florin Georgescu, Razvan Matei Bratu, Cristina Mambet, Valentin Enache, Mihaela Gheorghiu, Daniela Pasarica, Camelia Cristina Diaconu, Carmen Cristina Diaconu, Coralia Bleotu

**Affiliations:** 1 Faculty of Medicine, University of Medicine and Pharmacy Carol Davila Bucharest, 050474 Bucharest, Romania; vladalexandru.ionescu92@gmail.com (V.-A.I.); drgheorghe.gina@gmail.com (G.G.); claudiu.turculet@umfcd.ro (C.S.T.); florin.georgescu@umfcd.ro (T.F.G.); razvan.bratu@umfcd.ro (R.M.B.); cristina.mambet@gmail.com (C.M.); 2 Internal Medicine Department, Clinical Emergency Hospital of Bucharest, 105402 Bucharest, Romania; 3 General Surgery Department, Clinical Emergency Hospital of Bucharest, 105402 Bucharest, Romania; 4 Department of Cellular and Molecular Pathology, Stefan S. Nicolau Institute of Virology, Romanian Academy, 030304 Bucharest, Romania; coralia.bleotu@virology.ro; 5 Hematology Department, Emergency University Clinical Hospital of Bucharest, 050098 Bucharest, Romania; 6 Department of Anatomical Pathology, Clinical Emergency Hospital of Bucharest, 105402 Bucharest, Romania; valienache00@gmail.com; 7 Pathophysiology and Immunology Department, University of Medicine and Pharmacy Carol Davila Bucharest, 050474 Bucharest, Romania; mihaela.gheorghiu@umfcd.ro (M.G.); daniela.pasarica@umfcd.ro (D.P.); 8 Academy of Romanian Scientists, 050085 Bucharest, Romania; 9 Research Institute of the University of Bucharest (ICUB), University of Bucharest, 060023 Bucharest, Romania

**Keywords:** colorectal cancer, PDGF-AA, sTNF-R, IL-17A, TNF-α, IL-1β

## Abstract

Colorectal cancer (CRC) remains one of the most important causes of cancer-related mortality worldwide, underscoring the need to better understand systemic inflammatory pathways across the colorectal neoplasia spectrum. In this exploratory case–control study, we characterized plasma levels of key inflammatory mediators in healthy individuals and patients with colorectal polyps or CRC. Healthy controls (*n* = 10), patients with colorectal polyps (CP, n = 16), early-onset CRC (EO-CRC, n = 11), and late-onset CRC (LO-CRC, n = 51) were prospectively enrolled. Plasma levels of sTNF-R, total TNF-α, PDGF-AA, IL-17A, and IL-1β were measured by ELISA. Group comparisons used Kruskal–Wallis tests with epsilon-squared effect sizes. PDGF-AA showed the strongest differences between controls and all neoplastic groups (ε^2^ ≥ 0.15), and these comparisons remained significant after Benjamini–Hochberg false discovery rate correction. IL-17A levels were slightly higher in EO-CRC than in LO-CRC; however, this difference did not remain significant after adjustment for multiple testing. TNF-α and IL-1β showed no significant differences across groups. Overall, this study primarily provides descriptive and hypothesis-generating evidence of differential inflammatory patterns across colorectal neoplasia, with PDGF-AA emerging as the most robust signal in this exploratory dataset. These findings do not support immediate diagnostic application and require validation in larger, prospectively recruited cohorts.

## 1. Introduction

Colorectal cancer (CRC) remains one of the leading causes of morbidity and mortality worldwide. According to the International Agency for Research on Cancer, approximately 1.9 million new cases and 900,000 CRC-related deaths are reported globally each year [1]. An emerging concern highlighted in recent research is the rising incidence of CRC among individuals aged ≤50 years, a distinct entity known as early-onset CRC (EO-CRC) [2,3]. Moreover, patients with EO-CRC exhibit unique molecular and histopathological characteristics compared with those diagnosed after the age of 50—referred to as late-onset CRC (LO-CRC)—features that may contribute to their less favorable prognosis [3,4].

Given these observations, recent studies have focused on elucidating the pathophysiological mechanisms underlying CRC development, as well as identifying novel tools that could facilitate early diagnosis and improve therapeutic management. In this context, the accessibility and minimally invasive nature of peripheral blood-based testing have generated considerable interest [5,6,7]. Among the various pathways implicated in carcinogenesis, the inflammatory pathway has drawn particular attention. Numerous studies have described the cellular and molecular roles of inflammation in tumour initiation and progression [8,9,10]. However, the mechanisms through which chronic inflammation promotes tumorigenesis are not fully understood [11]. Accumulation of inflammatory cells and mediators within the tumour microenvironment (TME) stimulates malignant cell proliferation, metastasis, angiogenesis, epithelial–mesenchymal transition and may attenuate adaptive immune responses [11]. Tumour-associated inflammation has also been shown to influence clinical outcomes in CRC, involving both local immune responses and systemic inflammation [12,13]. For example, the composition of tumour-infiltrating lymphocytes within the TME correlates with prognosis [14], while circulating cytokines and systemic inflammatory markers have been proposed as potential diagnostic and prognostic biomarkers [12,15].

Among these mediators, interleukin-1 beta (IL-1β) and tumor necrosis factor alpha (TNF-α) activate nuclear factor kappa-light-chain-enhancer of activated B cells (NF-κB) and signal transducer and activator of transcription 3 (STAT3) pathways, promoting malignant cell invasion and angiogenesis [16]. Members of the tumor necrosis factor receptor superfamily (TNF-R) have emerged as key regulators of tumorigenesis, tumour progression, and treatment response [17,18]. Platelet-derived growth factor (PDGF), a potent regulator of cell proliferation and division, has also been implicated in uncontrolled angiogenesis across multiple solid tumors, including CRC [19,20,21,22]. Additionally, interleukin-17A (IL-17A) contributes to colorectal carcinogenesis by enhancing tumour cell invasion via activation of the phosphoinositide 3-kinase/protein kinase B/nuclear factor kappa-light chain enhancer of activated B cells (PI3K/AKT/NF-κB) signaling pathway and upregulation of matrix metalloproteinases MMP-2 and MMP-9 [23,24].

The present study aims to investigate the relationship between systemic inflammatory mediators and CRC by analyzing plasma levels of five cytokines with established roles in tumour biology: soluble TNF receptor (sTNF-R), total TNF-α, PDGF-AA, IL-17A, and IL-1β. These cytokines were selected because they represent key and complementary components of pro-tumorigenic inflammatory signaling: IL-1β and TNF-α are central activators of NF-κB/STAT3 pathways, sTNF-R provides a stable systemic indicator of TNF-mediated inflammation, PDGF-AA reflects stromal activation and angiogenesis, while IL-17A captures the Th17-driven axis associated with tumour invasion. The originality of this work lies in the comparative evaluation of these biomarkers across four distinct groups: healthy controls, patients with colorectal polyps (CP), individuals with EO-CRC, and those with LO-CRC. The primary objective is to identify expression differences that may distinguish early from late stages of colorectal carcinogenesis, as well as the specific inflammatory signatures associated with EO-CRC compared with LO-CRC. In addition, exploratory multivariable analyses based on a reduced cytokine panel were performed to assess whether multi-marker inflammatory signatures may provide complementary discriminative information beyond individual cytokines. Given the limited sample size, these analyses are intended to be hypothesis-generating.

## 2. Materials and Methods

### 2.1. Study Design and Population

We conducted a prospective, exploratory, case–control study over a 3-year interval (February 2023–October 2025) at the Clinical Emergency Hospital of Bucharest. Four study groups were defined:

(1) 10 healthy controls, with no colorectal lesions confirmed by colonoscopy;
(2) 16 patients with colorectal adenomatous polyps;
(3) 11 patients with early-onset colorectal cancer (EO-CRC, ≤50 years);
(4) 51 patients with late-onset colorectal cancer (LO-CRC, >50 years).

The study was designed to include a small cohort of healthy controls without colorectal lesions for comparative cytokine profiling. All controls self-reported absence of gastrointestinal symptoms, chronic inflammatory or autoimmune disease, recent infection, or known malignancy. At enrolment, participants were clinically screened and considered disease-free for study purposes. In the final enrolled sample, all individuals who met the eligibility criteria and agreed to participate were healthcare workers.

We note that none of the patients included in the study had a personal or family history of genetic syndromes associated with an increased risk of colorectal cancer (such as familial adenomatous polyposis, Lynch syndrome, etc.).

All participants signed informed consent for biological sampling and clinical data use. The study adhered to the Declaration of Helsinki and received approval from the institutional ethics committee (Approval No. 1400/7 February 2023).

Inclusion criteria: signed informed consent; histopathological diagnosis of CRC or colorectal adenomatous polyps; absence of malignancy at another site in the last 5 years; absence of recent infectious diseases or autoimmune disorders.

Exclusion criteria: lack of consent; synchronous malignancies; recent infections; autoimmune diseases. Two patients met exclusion criteria and were removed.

Demographic, clinical, and paraclinical data were recorded in a standardized Excel database. From each participant, 6 mL of peripheral blood were collected in two EDTA tubes and transported within 2 h to the Ștefan Nicolau Institute of Virology. Samples were centrifuged at 3500 rpm for 20 min, and plasma was aliquoted into Eppendorf tubes, coded, and stored at −80 °C until batch analysis.

### 2.2. Cytokine Quantification by ELISA

Plasma concentrations of sTNF-R (80 kDa), total TNF-α, PDGF-AA, IL-17A, and IL-1β were measured using commercial sandwich ELISA kits (ThermoFisher Scientific, Waltham, MA, USA), as follows: Human sTNF-R (80 kDa) Platinum ELISA, BMS211/BMS211TENCE, Human TNF-α KHC3011/KHC3012/KHC3011C, Human PDGF-AA ELISA EHPDGFA, Human IL-17A (homodimer) Uncoated ELISA 88-7176, Human IL-1β/IL-IF2 Immunoassay DLB50/SLB50/PDLB50. Prior to analysis, plasma samples were equilibrated to room temperature and centrifuged to remove debris. Where required (e.g., IL-17A), plates were pre-coated overnight at 4 °C with capture antibody and blocked using the supplied diluent. Standard curves were generated by 1:2 serial dilutions of reconstituted standards (e.g., IL-17A: 4–500 pg/mL; total TNF-α: 23–1500 pg/mL; sTNF-R: two-fold serial dilutions). Samples and standards were pipetted in duplicate (50–100 µL/well) and incubated for 2 h at room temperature on a microplate shaker. Plates were washed 3–6 times with PBS-Tween (PBS-T) according to kit specifications. Detection was carried out using biotin-labeled antibodies (IL-17A, TNF-α) or horseradish peroxidase (HRP)-conjugated antibodies (sTNF-R), followed by incubation with streptavidin-HRP or avidin-HRP. Colorimetric development was performed using tetramethylbenzidine (TMB) substrate, and reactions were stopped with acidic stop solution once the upper standard reached optimal optical density (OD ~0.9–0.95 for TNF-α). Absorbance was read at 450 nm (with 570/620 nm correction where applicable). The lower limits of detection (LOD) for the plasma cytokine assays were as follows: 0.10 ng/mL for sTNF-R (80 kDa), 1.7 pg/mL for total TNF-α, 40 pg/mL for PDGF-AA, 2 pg/mL for IL-17A, and 1 pg/mL for IL-1β. Values below the assay limit of detection were recorded as not detectable (ND); for statistical analysis, ND values were coded as 0. Cytokine levels were calculated via five-parameter logistic regression (5-PL), applying appropriate dilution factors (e.g., 1:10 for sTNF-R; 1:2 for total TNF-α). All measurements were performed in duplicate, and samples with >20% intra-duplicate variation were repeated.

### 2.3. Statistical Analysis

Statistical analysis was performed using R, version 4.3.2. Distribution of continuous variables was assessed visually (histograms, Q-Q plots) and by normality tests. Due to predominantly non-parametric distributions, data are presented as median (IQR), with minimum and maximum values.

To compare epidemiological, clinical, and paraclinical characteristics across the three patient groups (CP, EO-CRC, and LO-CRC), statistical tests were selected according to the nature of each variable. Continuous variables (e.g., age, body mass index—BMI) were analyzed using the Kruskal–Wallis test due to their non-normal distribution, and results were reported as median values with IQR. Nominal categorical variables (e.g., sex, tumour location, comorbidities, alcohol consumption, smoking status, family history of cancer) were compared using the Chi-square test (χ^2^). In cases where expected cell frequencies were <5 or distributions were markedly unbalanced—particularly within the EO-CRC group—Fisher’s exact test was employed as an alternative.

Comparisons of cytokine levels across groups (Control, CP, EO-CRC, LO-CRC) were conducted using the Kruskal–Wallis test. Effect size was calculated using epsilon-squared (ε^2^) according to the formula: ε^2^ = (H − k + 1)/(n − k), where H is the Kruskal–Wallis statistic, k the number of groups, and n the total sample size. Effect sizes were interpreted as small (<0.01), moderate (0.01–0.04), or large (>0.04). To account for multiple testing in the pairwise cytokine comparisons, *p*-values were adjusted using the Benjamini–Hochberg false discovery rate procedure. Because this study was exploratory and hypothesis-generating, pairwise findings were interpreted cautiously and alongside effect sizes rather than as confirmatory evidence.

To explore the apparent discriminatory ability of multimarker cytokine combinations, we constructed separate exploratory multivariable logistic regression models using a reduced three-cytokine panel consisting of sTNF-R, PDGF-AA and IL-17A. These cytokines were selected a priori based on their relatively larger between-group differences and to limit the number of predictors relative to sample size. For each comparison (controls vs. CP, controls vs. EO-CRC, controls vs. LO-CRC, CP vs. EO-CRC, CP vs. LO-CRC, and EO-CRC vs. LO-CRC), the three cytokines were entered as explanatory variables, and the resulting linear predictor was used for ROC curve analysis. Model performance was summarized using the area under the ROC curve (AUC), which in this context reflects only nominal, apparent discrimination. To avoid overemphasizing these exploratory analyses in the main text, the full ROC results are presented in the Supplementary Materials.

To quantify overfitting in the three-cytokine multivariable logistic regression models (sTNF-R, PDGF-AA and IL-17A), we performed bootstrap internal validation (B = 2000 resamples) for each pairwise comparison. In each resample, the model was refit and AUC was computed both in the bootstrap sample (apparent performance) and in the original dataset (test performance). Optimism was estimated as the mean difference AUC_bootstrap − AUC_test, and an optimism-corrected AUC was obtained by subtracting the estimated optimism from the apparent AUC.

Given the limited sample size, small number of events in some subgroups, and unbalanced group structure, these multivariable models remain at substantial risk of overfitting despite internal bootstrap validation. Accordingly, all ROC analyses should be regarded as strictly exploratory; the reported AUC values represent nominal, hypothesis-generating estimates rather than definitive measures of diagnostic performance and should not be interpreted as evidence of clinical utility.

## 3. Results

In the first part of the study, we performed a descriptive analysis to explore potential statistically significant differences among the three patient groups. The median age of patients with CP was 69.5 years, compared with 45 years in the EO-CRC group and 71 years in the LO-CRC group (Table 1). The differences in age were statistically significant, an expected finding given that age is inherently part of the definition of EO-CRC and LO-CRC; nonetheless, the inclusion of the CP group justified the comparative statistical analysis (Table 1). Regarding sex distribution, we observed a slight predominance of females in the first two groups and a slight predominance of males in the LO-CRC group. However, these differences were not statistically significant (χ^2^ = 2.30, df = 2, *p* = 0.316), and any subgroup-level descriptive differences should be interpreted cautiously given the limited size of the EO-CRC cohort.

Comparison of clinical presentation across the three groups revealed no statistically significant differences (χ^2^ = 18.97, df = 12, *p* = 0.089), although certain symptoms were more frequent in specific groups (anemia in CP, rectal bleeding in EO-CRC, and abdominal pain in LO-CRC) (Table 1). In contrast, the anatomical distribution of lesions differed significantly between groups (χ^2^ = 13.20, df = 2, *p* = 0.010). Patients with CP presented predominantly left-sided lesions, those with EO-CRC appeared to show a mainly distal distribution, whereas LO-CRC patients showed the expected higher proportion of right-sided tumors (Table 1).

For comparative analyses of endoscopic appearance, histopathological diagnosis, tumor grade, and tumor stage, the CP group was excluded, as these parameters are either not applicable or differ fundamentally between benign and malignant lesions. Concerning endoscopic appearance, no statistically significant differences were identified between EO-CRC and LO-CRC patients (χ^2^ = 0.77, df = 2, *p* = 0.380). Histopathological diagnosis also did not differ significantly between the two groups (χ^2^ = 1.54, df = 3, *p* = 0.674), with most tumors being adenocarcinoma not otherwise specified (AC-NOS) and mucinous subtypes being rare (Table 1). Similarly, tumor grade did not differ significantly, with the majority of tumors being moderately differentiated (χ^2^ = 2.88, df = 2, *p* = 0.237). However, tumor stage differed considerably between groups. EO-CRC patients appeared to show a substantially higher frequency of stage IV disease compared with LO-CRC patients (45.5% vs. 15.7%). Given the small sample size and uneven distribution of stages in the EO-CRC group, Fisher’s exact test was applied as an additional analysis, confirming the statistical significance of this difference (OR = 4.48, *p* = 0.043). This subgroup-level observation should nevertheless be interpreted cautiously and regarded as preliminary.

Median BMI was slightly higher in the CP group (28.23 kg/m^2^, IQR 26.0–33.6) compared with EO-CRC (25.39 kg/m^2^, IQR 23.5–29.28) and LO-CRC (25.55 kg/m^2^, IQR 22.4–29.98), although the difference was not statistically significant (Kruskal–Wallis H = 1.89, df = 2, *p* = 0.392).

Smoking status differed significantly among the three groups (χ^2^ = 11.63, df = 2, *p* = 0.003). The highest proportion of smokers was observed in the CP group (75%), followed by EO-CRC (63.6%) and LO-CRC (33.3%). In contrast, no statistically significant differences were identified between groups in terms of alcohol consumption, statin or aspirin use, hypertension, diabetes mellitus, dyslipidemia, history of cholecystectomy, or family history of cancer.

**Figure 1:**
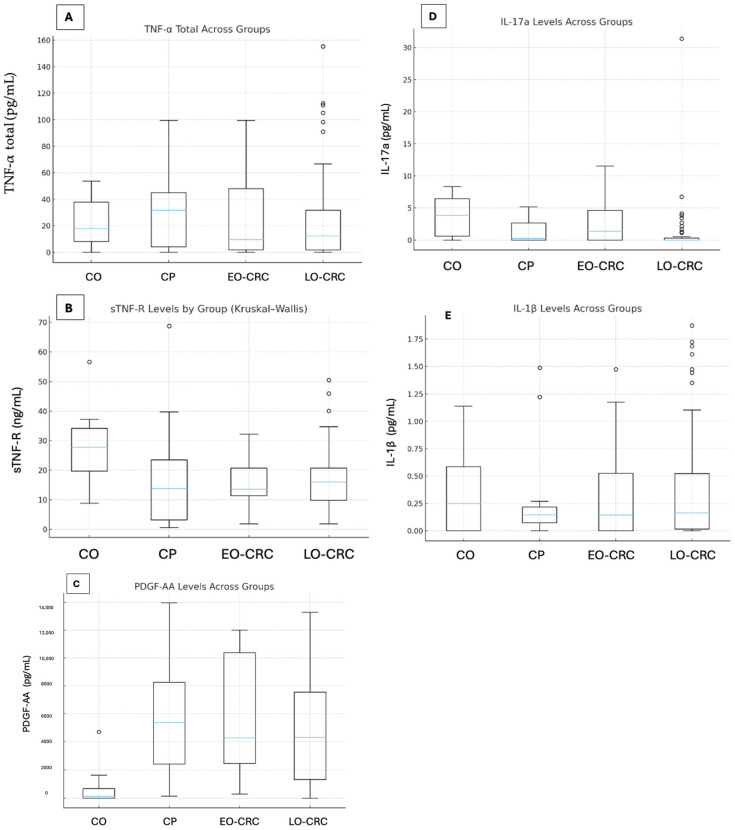
Distribution of plasma cytokine concentrations across study groups—healthy controls (CO), patients with colorectal polyps (CP), early-onset colorectal cancer (EO-CRC), and late-onset colorectal cancer (LO-CRC). Overall group differences were assessed using the Kruskal–Wallis test. Panels: (**A**) total tumor necrosis factor alpha—TNF-α; (**B**) soluble TNF receptor—sTNF-R; (**C**) platelet-derived growth factor—PDGF; (**D**) interleukin-17A—IL-17A; (**E**) interleukin-1 beta—IL-1β.

Plasma concentrations of the five analyzed cytokines exhibited substantial variability across the four study groups. Median values, interquartile ranges (IQR), and minimum and maximum concentrations for each group are summarized in Table 2. For sTNF-R, the highest median plasma levels were observed in the control group, whereas lower median concentrations were recorded in patients with CP, EO-CRC, and LO-CRC. The distributions illustrated in Figure 1 demonstrate a general downward shift in sTNF-R values in pathological groups compared with controls. Total TNF-α concentrations exhibited substantial dispersion within all groups, with wide IQRs and overlapping ranges. The highest median TNF-α value was observed in the colorectal polyp group; however, marked intra-group variability was present, and no clear separation between groups was evident based on distribution plots. PDGF-AA concentrations differed more markedly between groups. Median PDGF-AA levels were substantially higher in all pathological groups compared with controls, with limited overlap between control values and those observed in patients with CP and CRC. The distributions across the pathological groups were broadly similar. IL-17A and IL-1β displayed low median plasma concentrations across all study groups, with values clustered near the lower end of the detection range. Their distributions showed extensive overlap between groups, as illustrated in Figure 1, and no clear group-specific separation was observed. Any apparent differences involving EO-CRC should be interpreted cautiously, given the small and uneven subgroup sizes, and are best regarded as preliminary descriptive observations within an exploratory dataset.

Figure 1 illustrates the distribution of each cytokine across the study groups, highlighting both intergroup variability and the presence of extreme values. In addition, the graphical representations emphasize the substantial overlap between groups for several cytokines, as well as the heterogeneity of individual measurements within each cohort, underscoring the absence of clear distributional cut-offs between disease categories.

The pairwise comparisons performed between study groups for all five cytokines are summarized in Table 3. Based on nominal *p*-values, the most evident between-group differences were observed for sTNF-R, PDGF-AA, and IL-17A. However, after Benjamini–Hochberg false discovery rate correction for multiple testing, the most robust findings were retained for PDGF-AA in all control-versus-pathology comparisons (CO vs. CP, CO vs. EO-CRC, and CO vs. LO-CRC) and for IL-17A in the comparison between controls and LO-CRC. For sTNF-R, healthy controls exhibited higher plasma levels compared with all pathological groups, with effect sizes ranging from medium to large, indicating consistent between-group differences. However, these nominally significant differences did not remain significant after FDR correction. Within the pathological groups, patients with LO-CRC showed slightly higher sTNF-R concentrations compared with those with CP and EO-CRC; however, these differences did not reach statistical significance (Table 2 and Table 3). For total TNF-α, none of the pairwise comparisons reached statistical significance, and epsilon^2^ values were zero, suggesting the absence of a meaningful effect across groups. In contrast, PDGF-AA demonstrated pronounced differences between controls and all pathological groups, consistently accompanied by large effect sizes (epsilon^2^ ≥ 0.15). These findings support an association between elevated PDGF-AA levels and colorectal neoplastic pathology. Nevertheless, no statistically significant differences were identified among the pathological groups (Table 3). Regarding IL-17A, control subjects displayed slightly higher serum levels compared with patients with LO-CRC, and this difference remained significant after FDR correction (Table 3). When comparing pathological subgroups, EO-CRC patients exhibited marginally higher IL-17A levels than those with LO-CRC, a statistically significant difference but with a small-to-medium effect size (Table 3). However, this nominally significant difference did not remain significant after adjustment for multiple testing. Given the small and uneven subgroup sizes, particularly for EO-CRC, this finding should be interpreted cautiously and regarded as preliminary and hypothesis-generating rather than as evidence of a definitive subtype-specific inflammatory pattern. For IL-1β, no statistically significant differences were observed across any of the group comparisons, and epsilon^2^ values consistently indicated the absence of an effect in all analyses (Table 3).

**Figure 2:**
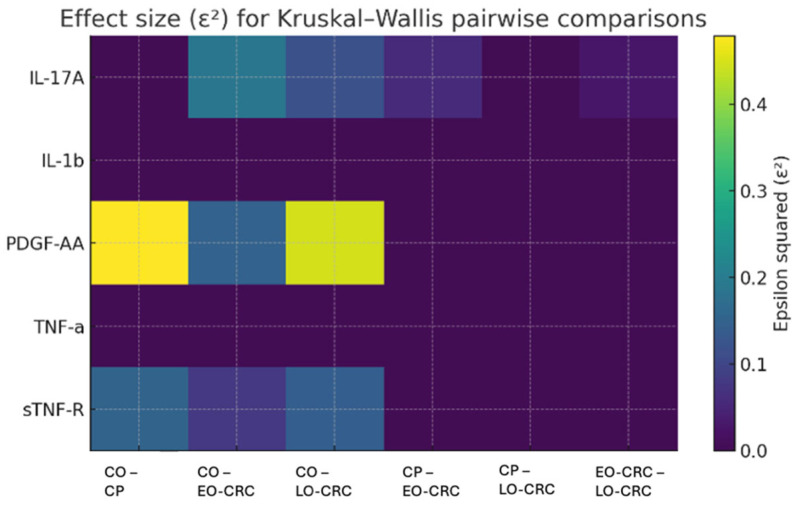
Effect size (ε^2^) for pairwise nonparametric comparisons between the study groups. Abbreviations: healthy controls—CO, patients with colorectal polyps—CP, early-onset colorectal cancer—EO-CRC, late-onset colorectal cancer—LO-CRC, tumor necrosis factor alpha—TNF-α, soluble TNF receptor—sTNF-R, platelet-derived growth factor—PDGF, interleukin-17A—IL-17A, interleukin-1 beta—IL-1b.

Figure 2 illustrates the epsilon-squared effect sizes associated with the pairwise comparisons of plasma cytokine concentrations across the study groups. The largest effect sizes were observed for PDGF-AA in comparisons involving the control cohort, suggesting pronounced differences between individuals without neoplastic pathology and those with colorectal neoplastic conditions.

Exploratory multivariable ROC analyses based on a reduced three-cytokine panel (sTNF-R, PDGF-AA and IL-17A) were performed to assess whether combined cytokine patterns might provide preliminary signal-detection across study groups. However, given the small and unbalanced cohort size, the events-per-variable criterion was not met, particularly in subgroup comparisons, making overfitting likely. Although apparent performance was observed in some control-versus-pathology comparisons, bootstrap correction attenuated these estimates, and subgroup analyses showed limited and unstable separation. To avoid overinterpretation of apparent model performance in this small selected dataset, the full ROC analyses are presented in the Supplementary Materials and should be regarded as exploratory and hypothesis-generating rather than as evidence of clinical utility.

## 4. Discussion

In this exploratory study, we investigated the systemic inflammatory profile associated with different stages of colorectal neoplasia by quantifying the plasma levels of five cytokines with well-established roles in carcinogenesis: sTNF-R, total TNF-α, PDGF-AA, IL-17A, and IL-1β. Our findings reveal distinct cytokine expression patterns across healthy controls and patients with CP, EO-CRC and LO-CRC, highlighting both expected and paradoxical trends that may provide new insights into the complex interplay between systemic inflammation and colorectal tumorigenesis. A secondary objective of the study was to comparatively assess clinical and paraclinical characteristics among patients with CP, EO-CRC, and LO-CRC.

We identified statistically significant differences among the three groups with respect to tumour location, tumour stage, and smoking status. CP cases most frequently exhibited left-sided lesions (81.2%), EO-CRC appeared to show a predominantly distal distribution (36.4% left colon, 36.4% rectum), whereas LO-CRC included a higher proportion of right-sided tumours (43.1%). These observations are broadly consistent with the existing literature, although the EO-CRC subgroup in the present study was small and therefore these findings should be interpreted with caution. Zhu et al. recently reported the sigmoid colon as the most common site of sporadic colorectal polyps, followed by the transverse colon [25]. Likewise, previous studies have documented a tendency toward distal tumour location in EO-CRC and proximal location in LO-CRC [3,26]. Moreover, EO-CRC patients in our cohort appeared to have a higher prevalence of smoking and more advanced disease stages, in concordance with prior reports [3,27,28]. However, given the limited number of EO-CRC cases, these subgroup-level observations should be regarded as preliminary and hypothesis-generating rather than definitive.

Among all cytokines analysed, PDGF-AA showed the most pronounced differences between controls and all pathological groups. This is consistent with previous evidence implicating PDGF-AA in stromal activation, tumour–stroma interactions, endothelial proliferation, and aberrant angiogenesis in CRC [29,30]. The large effect sizes observed (ε^2^ ≥ 0.15) support the notion that PDGF-AA represents a robust marker even for early precancerous lesions such as CP. Notably, PDGF-AA concentrations did not differ between EO-CRC and LO-CRC, suggesting that its upregulation reflects carcinogenic processes shared by both subtypes rather than age-specific inflammatory signatures. Importantly, after Benjamini–Hochberg false discovery rate correction, PDGF-AA retained significance in all control-versus-pathology comparisons, supporting the robustness of this finding within the exploratory framework of the present study.

sTNF-R concentrations were higher in healthy controls than in all neoplastic groups. However, this finding did not remain statistically significant after false discovery rate correction. CRC has been reported to promote an immunosuppressive systemic milieu, characterized by T-cell exhaustion, expansion of myeloid-derived suppressor cells, and diminished responsiveness to pro-inflammatory cytokines, all of which may attenuate TNF-α/TNF-R signalling [18,31,32,33]. Nevertheless, in the present study, this observation should be interpreted with caution, particularly given the small sample size.

By contrast, IL-17A concentrations were higher in controls than in patients with LO-CRC, and this difference remained statistically significant after FDR correction. The role of IL-17A in cancer remains controversial in the literature. Some studies support a pro-tumour role through the promotion of angiogenesis and inflammation, whereas others suggest a possible anti-tumour role, including the recruitment of CD8^+^ T cells within the tumour microenvironment [34,35,36]. In our study, the apparent elevation of IL-17A in controls should be viewed primarily as a methodological uncertainty that constrains biological interpretation and underscores the need for larger, population-based studies with more representative control groups before any firm biological or translational inferences can be made.

TNF-α and IL-1β showed no significant differences between study groups. Both cytokines are rapidly degraded in circulation, secreted in a pulsatile manner, and highly sensitive to pre-analytical conditions, which likely explains the absence of significant differences [37,38]. By contrast, sTNF-R has a longer half-life and greater stability, offering a plausible explanation for the significant differences detected for sTNF-R but not for TNF-α [39].

IL-17A exhibited modest but statistically significant differences across groups, with EO-CRC patients showing slightly higher levels compared with LO-CRC. This observation may be of interest in light of the findings of Albujassim et al., who reported that elevated IL-17A in CRC correlates with advanced tumour stage and poorer prognosis [40]. However, given the small and uneven subgroup sizes, particularly for EO-CRC, this finding should be interpreted cautiously and regarded as preliminary and hypothesis-generating rather than as evidence of a definitive subtype-specific inflammatory signature. Accordingly, the present data may suggest, but do not establish, that IL-17A could contribute to the more aggressive clinical behaviour observed in EO-CRC. Notably, however, the nominally significant EO-CRC versus LO-CRC difference did not remain significant after adjustment for multiple testing. Conversely, and somewhat unexpectedly, healthy controls displayed slightly higher IL-17A concentrations than all pathological groups. This pattern should also be interpreted with caution. Although some studies have suggested a potential anti-tumour role for IL-17A in specific immunological settings [41,42], the composition of the control cohort and the small sample size in the present study limit biological inference. In addition, chronic stress has been associated with increased circulating IL-17A levels in multiple studies [43,44,45,46]. Within this context, stress-related or other control-specific factors may have contributed to the relatively higher IL-17A levels observed in controls. Nevertheless, this interpretation remains speculative, and the present findings should not be taken as evidence of a protective inflammatory profile. Among the IL-17A pairwise comparisons, only the difference between controls and LO-CRC remained significant after false discovery rate correction. Larger, mechanistic and population-based studies are required to clarify the role of IL-17A across colorectal neoplastic states.

A secondary aim of our study was to explore the possible signal-detection value of multimarker inflammatory cytokine combinations. Using a reduced three-cytokine panel (sTNF-R, PDGF-AA and IL-17A), exploratory multivariable models showed apparent separation between healthy controls and pathological cohorts, whereas subgroup discrimination was modest and unstable after bootstrap correction. Because recommended events-per-variable criteria were not met and validation was limited to internal bootstrap procedures, these analyses were considered highly susceptible to overfitting and are therefore presented in the Supplementary Materials. Accordingly, they should be regarded strictly as hypothesis-generating proof-of-concept findings and not as evidence of clinical utility.

The main limitations of this work relate to the relatively small and uneven subgroup sizes, particularly for EO-CRC, as well as the composition of the control cohort. Given that EO-CRC accounts for only ~11% of CRC cases [47,48,49], small EO-CRC subsamples are common in exploratory studies; nevertheless, the recommended events-per-variable criteria were not met, and internal bootstrap corrections indicate a risk of overfitting in multivariable analyses. An additional limitation is that, although the study was designed to include healthy controls without colorectal lesions, all enrolled controls happened to be healthcare workers. This may have introduced selection bias and may have influenced baseline inflammatory marker levels through unmeasured occupational, behavioural, or lifestyle factors. At the same time, a clinically screened, disease-free control group remains informative as an asymptomatic comparator in a hypothesis-generating setting. Validation in larger, population-based cohorts with more representative control populations is required to confirm these findings and to better account for clinical, behavioural, and lifestyle confounders.

In interpreting the observed cytokine patterns, potential confounding by tumour stage, smoking status, BMI, comorbidities, and medication use should also be considered. These factors may have influenced circulating cytokine levels independently of neoplasia status or age of onset and may therefore have contributed to the observed between-group differences. Because of the small and uneven subgroup sizes, particularly for EO-CRC and controls, formal multivariable adjustment for these potential confounders was not considered statistically reliable in the present dataset and may have introduced additional model instability. In addition, values below the assay limit of detection were coded as 0 for statistical analysis. Although this allowed consistent handling of low or undetectable measurements, it may have affected distributional comparisons and effect estimates, particularly for analytes concentrated near the lower detection range.

A further limitation is the lack of an external validation cohort. Confirmation of the present findings in an independent population would require a separate validation study and remains an important objective for future research. Future studies should also incorporate longitudinal cytokine profiling to capture temporal inflammatory dynamics, integrate cytokine signatures with genomic and microbiome data, refine multi-marker models using modern statistical or machine-learning approaches, and prospectively evaluate such cytokine panels as exploratory adjunct biomarkers within precision CRC risk-stratification frameworks rather than as standalone screening tools.

## 5. Conclusions

In this exploratory study, we identified differences in circulating inflammatory mediators across distinct stages of colorectal neoplasia. Among the cytokines analysed, PDGF-AA showed the most consistent elevation in pathological groups, in line with its known role in stromal activation and angiogenic signalling. After adjustment for multiple testing, PDGF-AA remained the most robust marker, retaining significance in all control-versus-pathology comparisons. By contrast, higher sTNF-R and IL-17A concentrations in healthy controls underscore the methodological and biological uncertainty inherent to small exploratory studies and highlight the influence of study design, control group characteristics, and non-neoplastic inflammatory processes on cytokine quantification. Taken together, these findings suggest that systemic inflammatory signalling may reflect aspects of colorectal tumourigenesis, but the unexpected cytokine patterns observed in controls and the small subgroup sizes limit inference. The present work should therefore be viewed as primarily descriptive and hypothesis-generating, requiring validation in larger, population-based cohorts before any clinical or translational implications can be considered.

Exploratory multimarker analyses, presented in the Supplementary Materials, suggested apparent separation between healthy controls and pathological groups, but subgroup discrimination was modest and unstable after bootstrap correction. Because subgroup sizes were small and the recommended events-per-variable criteria were not met, these multimarker findings should also be regarded strictly as hypothesis-generating and should not be interpreted as evidence of predictive or diagnostic utility at this stage.

## Figures and Tables

**Table 1:** Baseline Characteristics of Study Cohorts. Group comparisons were performed using the Kruskal–Wallis test for continuous variables and the Chi-square or Fisher’s exact test, as appropriate, for categorical variables. Abbreviations: CO-control, CP—colorectal polyps, EO-CRC—early-onset colorectal cancer, LO-CRC—late-onset colorectal cancer, min—minimum, max—maximum, AC-NOS—adenocarcinoma not otherwise specified, MAC—mucinous adenocarcinoma, CIS—carcinoma in situ, G1—well differentiated, G2—moderately differentiated, G3—poorly differentiated, BMI—body mass index, na—not applicable.

Parameter		CO	CP (n = 16)	EO-CRC (n = 11)	LO-CRC (n = 51)	*p* Value
Age (years)	Median age	52	69.5	45	71	<0.0001
	Min	32	38	25	55	
	Max	57	79	50	87	
Sex distribution	Female	6 (60%)	11 (68.8%)	6 (54.5%)	24 (47.1%)	0.316
	Male	4 (40%)	5 (31.2%)	5 (45.5%)	27 (52.9%)	
Clinical presentation	Anemia	na	5 (31.2%)	1 (9.1%)	14 (27.5%)	0.089
	Melena	na	0 (0.0%)	1 (9.1%)	2 (3.9%)	
	Abdominal pain	na	4 (25.0%)	3 (27.3%)	17 (33.3%)	
	Rectal bleeding	na	4 (25.0%)	5 (45.5%)	12 (23.5%)	
	Chronic constipation	na	1 (6.2%)	0 (0.0%)	4 (7.8%)	
	Unintentional weight loss	na	1 (6.2%)	0 (0.0%)	2 (3.9%)	
	Screening	na	1 (6.2%)	1 (9.1%)	0 (0.0%)	
Tumor location	Right colon	na	3 (18.8%)	3 (27.3%)	22 (43.1%)	0.010
	Left colon	na	13 (81.2%)	4 (36.4%)	15 (29.4%)	
	Rectum	na	0 (0.0%)	4 (36.4%)	14 (27.5%)	
Endoscopic appearance	Sessile	na	7 (43.8%)	na	na	0.380
	Pedunculated	na	9 (56.2%)	na	na	
	Stenosing	na	na	4 (36.4%)	27 (52.9%)	
	Exophytic	na	na	7 (63.6%)	23 (45%)	
	Infiltrative	na	na	0 (0.0%)	1 (2.1%)	
Histopathological diagnosis	AC-NOS	na	na	10 (90.9%)	41 (78.8%)	0.674
	AC-NOS with mucinous component	na	na	0 (0.0%)	5 (9.6%)	
	MAC	na	na	1 (9.1%)	3 (5.8%)	
	CIS	na	na	0 (0.0%)	2 (3.8%)	
Tumor grade	G1-G2	na	na	1 (9.1%)	3 (5.9%)	0.237
	G2	na	na	10 (90.9%)	37 (72.5%)	
	G3	na	na	0 (0.0%)	9 (17.6%)	
	CIS	na	na	0 (0.0%)	2 (3.9%)	
Tumor stage	I	na	na	2 (18.2%)	8 (15.7%)	0.043
	II	na	na	2 (18.2%)	18 (35.3%)	
	III	na	na	2 (18.2%)	17 (33.3%)	
	IV	na	na	5 (45.5%)	8 (15.7%)	
Median BMI (kg/m^2^)		27.32	28.23	25.39	25.55	0.392
Smoker		4 (40%)	12 (75.0%)	7 (63.6%)	17 (33.3%)	0.003
Alcohol consumption		3 (30%)	4 (25%)	3 (27.3%)	14 (27.5%)	0.744
Statin use		0 (0.0%)	9 (56.2%)	4 (36.4%)	18 (35.3%)	0.292
Aspirin use		0 (0.0%)	5 (31.3%)	1 (9.1%)	10 (19.60%)	0.36
Cholecystectomy history		0 (0.0%)	4 (25%)	2 (18.2%)	3 (5.9%)	0.086
Family history of cancer		2 (20%)	2 (12.5%)	4 (36.4%)	15 (29.4%)	0.31
Diabetes mellitus		0 (0.0%)	6 (37.1%)	1 (9.1%)	10 (19.6%)	0.17
Hypertension		0 (0.0%)	10 (62.5%)	5 (45.5%)	29 (56.9%)	0.67
Dyslipidemia		0 (0.0%)	6 (37.5%)	2 (18.18%)	20 (39.2%)	0.42

**Table 2:** Plasma cytokine concentrations across study groups. Values are presented as median, IQR (25–75%), minimum, and maximum. Abbreviations: CO—control, CP—colorectal polyps, EO-CRC—early-onset colorectal cancer, LO-CRC—late-onset colorectal cancer, tumor necrosis factor alpha—TNF-α, soluble TNF receptor—sTNF-R, platelet-derived growth factor—PDGF, interleukin-17A—IL-17A, interleukin-1 beta—IL-1β. ND indicate measurements below the limits of detection.

Cytokine	Group	Median	IQR (25–75%)	Min	Max
sTNF-R (ng/mL)	CO	27.77	19.69–34.25	8.80	56.60
	CP	13.81	3.22–23.53	0.61	68.74
	EO-CRC	13.66	11.39–20.79	1.82	32.17
	LO-CRC	16.08	9.87–20.79	1.82	50.53
TNF-α total (pg/mL)	CO	18.14	8.14–37.79	ND	53.86
	CP	31.72	4.22–44.93	ND	99.57
	EO-CRC	9.57	1.93–48.14	ND	99.57
	LO-CRC	12.43	1.72–31.72	ND	155.29
PDGF-AA (pg/mL)	CO	112.5	0.00–680	ND	4720
	CP	5377.5	2428.75–8261.25	140	13,965
	EO-CRC	4295	2480–10,390	290	12,000
	LO-CRC	4329	1327–7557.5	ND	13,290
IL-17A (pg/mL)	CO	3.86	0.63–6.47	ND	8.35
	CP	0.26	0.00–2.66	ND	5.16
	EO-CRC	1.39	0.00–4.65	ND	11.54
	LO-CRC	0.00	0.00–0.305	ND	31.39
IL-1β (pg/mL)	CO	0.2485	0.00–0.5835	ND	1.14
	CP	0.1445	0.07175–0.2185	ND	1.488
	EO-CRC	0.143	0.00–0.526	ND	1.474
	LO-CRC	0.163	0.015–0.524	ND	1.873

**Table 3:** Pairwise nonparametric comparisons between study groups for all cytokines, including nominal *p*-values and Benjamini–Hochberg false discovery rate (FDR)-adjusted *p*-values. Abbreviations: tumor necrosis factor alpha—TNF-α, soluble TNF receptor—sTNF-R, platelet-derived growth factor—PDGF, interleukin-17A—IL-17A, interleukin-1 beta—IL-1β.

Cytokine	Comparison	H Statistic	*p* Value	FDR Adjusted *p*	Epsilon^2^	Effect Size Class
sTNF-R	CO vs. CP	4.446	0.0349	0.1539	0.1436	Large
	CO vs. EO-CRC	3.888	0.0486	0.1690	0.1520	Large
	CO vs. LO-CRC	5.696	0.0170	0.1020	0.0796	Medium
	CP vs. EO-CRC	0.049	0.824	0.9508	0	None
	CP vs. LO-CRC	1.090	0.296	0.8073	0.0014	Negligible
	EO-CRC vs. LO-CRC	0.150	0.699	0.9388	0	None
TNF-α total	CO vs. CP	0.2017	0.653	0.9388	0	None
	CO vs. EO-CRC	0.0012	0.972	0.9720	0	None
	CO vs. LO-CRC	0.2115	0.646	0.9388	0	None
	CP vs. EO-CRC	0.1207	0.728	0.9388	0	None
	CP vs. LO-CRC	0.9515	0.329	0.8225	0	None
	EO-CRC vs. LO-CRC	0.0014	0.970	0.9720	0	None
PDGF-AA	CO vs. CP	11.776	0.00060	0.0090	0.4490	Large
	CO vs. EO-CRC	10.107	0.00148	0.0123	0.4793	Large
	CO vs. LO-CRC	9.912	0.00164	0.0123	0.1511	Large
	CP vs. EO-CRC	0.0219	0.882	0.9720	0	None
	CP vs. LO-CRC	0.6793	0.410	0.8456	0	None
	EO-CRC vs. LO-CRC	0.8337	0.361	0.8331	0	None
IL-17A	CO vs. CP	3.818	0.0507	0.1690	0.1174	Medium
	CO vs. EO-CRC	0.568	0.451	0.8456	0	None
	CO vs. LO-CRC	12.024	0.000525	0.0090	0.1869	Large
	CP vs. EO-CRC	0.617	0.432	0.8456	0	None
	CP vs. LO-CRC	2.927	0.087	0.2610	0.0296	Small
	EO-CRC vs. LO-CRC	4.402	0.0359	0.1539	0.0567	Small-medium
IL-1β (pg/mL)	CO vs. CP	0.1007	0.751	0.9388	0	None
	CO vs. EO-CRC	0.0052	0.942	0.9720	0	None
	CO vs. LO-CRC	0.0513	0.821	0.9508	0	None
	CP vs. EO-CRC	0.1201	0.729	0.9388	0	None
	CP vs. LO-CRC	0.1475	0.701	0.9388	0	None
	EO-CRC vs. LO-CRC	0.2622	0.609	0.9388	0	None

## Data Availability

The original contributions presented in this study are included in the article/Supplementary Materials. Further inquiries can be directed to the corresponding author.

## Supplementary Materials

The following supporting information can be downloaded at: https://www.mdpi.com/article/10.3390/biom16030426/s1, Figure S1. Receiver operating characteristic (ROC) curves illustrating the apparent and optimism-corrected performance of a reduced three-cytokine plasma panel (sTNF-R, PDGF-AA and IL-17A) for distinguishing healthy controls (CO) from patients with colorectal polyps (CP), early-onset colorectal cancer (EO-CRC), and late-onset colorectal cancer (LO-CRC); Figure S2. Receiver operating characteristic (ROC) curves illustrating the apparent and optimism-corrected performance of a reduced three-cytokine plasma panel (sTNF-R, PDGF-AA and IL-17A) in differentiating colorectal polyps (CP) from early-onset colorectal cancer (EO-CRC) and late-onset colorectal cancer (LO-CRC), as well as in distinguishing EO-CRC from LO-CRC.
