# Supplementary material for: Differential Plasma Expression of sTNF-R, TNF-α, PDGF-AA, IL-17A, and IL-1β Across the Colorectal Neoplasia Spectrum
Source: Biomolecules. 2026 Mar 13;16(3):426. doi: 10.3390/biom16030426 (PMC13023994; doi:10.3390/biom16030426)
Supplement: Supplementary file 1 [file biomolecules-16-00426-s001.zip › biomolecules-4172975-supplementary.pdf]

Supplementary Materials

Results

Using a reduced three-cytokine panel consisting of sTNF-R, PDGF-AA and IL-17A—selected a priori based on their relatively larger between-group differences and to limit the number of predictors relative to sample size—the multivariable logistic regression model showed apparent performance between healthy controls and pathological cohorts. The apparent AUC values were 0.956 for CO vs. CP, 0.991 for CO vs. EO-CRC, and 0.882 for CO vs. LO-CRC (Figure S1). Following bootstrap internal validation (B = 2000) to quantify optimism, the corresponding optimism-corrected AUCs were 0.912, 0.941, and 0.848, respectively. Given the small and unbalanced group sizes, these ROC results should be interpreted strictly as exploratory and hypothesis-generating, rather than inferential or confirmatory. In addition, the events-per-variable criterion was not met,
indicating a substantial risk of overfitting; therefore, these analyses should not be interpreted as evidence of clinical utility. These exploratory ROC analyses are presented in the Supplementary Materials to avoid overinterpretation in the main text.

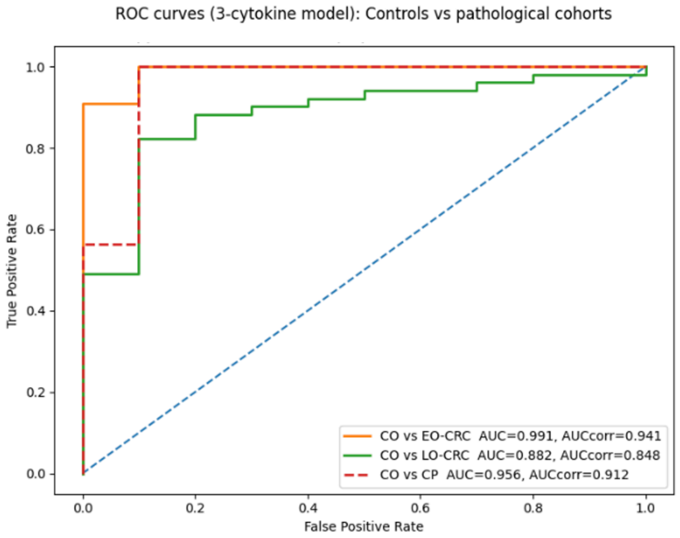

Figure S1. Receiver operating characteristic (ROC) curves illustrating the apparent and optimism-corrected performance of a reduced three-cytokine plasma panel (sTNF-R, PDGF-AA and IL-17A) for distinguishing healthy controls (CO) from patients with colorectal polyps (CP), early-onset colorectal cancer (EO-CRC), and late-onset colorectal cancer (LO-CRC). Apparent and optimism-corrected AUC values were obtained using internal bootstrap validation (B = 2000). Results are exploratory and hypothesis-generating and limited by likely overfitting.

When comparing pathological subgroups directly, the three-cytokine panel showed only modest apparent performance. The apparent AUC values were 0.653 for CP vs. EO-CRC, 0.622 for EO-CRC vs. LO-CRC, and 0.566 for CP vs. LO-CRC (Figure S2). Following bootstrap internal validation (B = 2000), the corresponding optimism-corrected AUCs were 0.517, 0.535, and 0.466, indicating substantial overfitting in these subgroup comparisons. These findings suggest only limited and unstable subgroup separation in this dataset, likely reflecting shared tumor-associated inflammatory pathways. Given that the events-per-variable criterion was not met and the small and unbalanced subgroups, these multivariable ROC analyses should be considered strictly exploratory and hypothesis-generating.

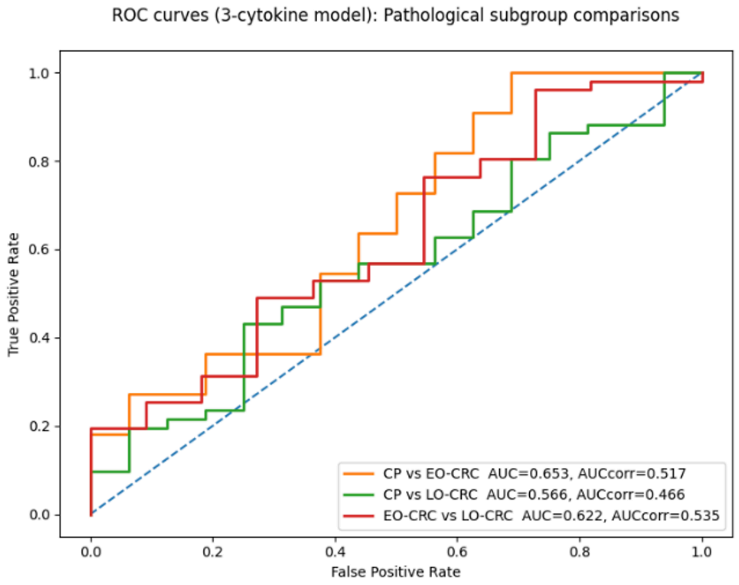

Figure S2. Receiver operating characteristic (ROC) curves illustrating the apparent and optimism-corrected performance of a reduced three-cytokine plasma panel (sTNF-R, PDGF-AA and IL-17A) in differentiating colorectal polyps (CP) from early-onset colorectal cancer (EO-CRC) and late-onset colorectal cancer (LO-CRC), as well as in distinguishing EO-CRC from LO-CRC. Apparent and bootstrap optimism-corrected AUC values (B = 2000) are shown. These subgroup analyses are exploratory and hypothesis-generating and limited by likely overfitting.
